# Grape seed proanthocyanidins improve lymphatic drainage and blood perfusion in secondary lymphedema models

**DOI:** 10.3389/fonc.2025.1553090

**Published:** 2025-06-06

**Authors:** Hwayeong Cheon, Bumchul Kim, Jae Yong Jeon

**Affiliations:** ^1^ Rehabilitation Research Center, Biomedical Engineering Research Center, Asan Institute for Life Sciences, Asan Medical Center, Seoul, Republic of Korea; ^2^ Department of Rehabilitation Medicine, Asan Medical Center, University of Ulsan College of Medicine, Seoul, Republic of Korea

**Keywords:** lymphedema, grape seed proanthocyanidin extract (GSPE), preclinical (*in vivo*) studies, lymphangiography, Doppler flowmetry, pharmacological treatment

## Abstract

**Introduction:**

Secondary lymphedema (SLE) is a chronic and debilitating condition that frequently arises following cancer treatments, particularly in breast cancer patients. Despite its increasing global prevalence and impact on patients’ quality of life, there remains no effective pharmacological treatment for SLE. Grape seed proanthocyanidin extract (GSPE), a compound known for treating venous insufficiency, is hypothesized to enhance lymphatic function and may offer therapeutic value for managing SLE. This study aimed to evaluate the efficacy of GSPE in a rat model of secondary lymphedema.

**Methods:**

Fifteen nine-week-old Sprague-Dawley rats (weighing 250–300 g) were used in this study. Tail lymphedema was surgically induced in 12 rats to model SLE, while 3 rats served as normal controls. The lymphedema-induced rats were randomly assigned to either a treatment group (n=6) or a control group (n=6). The treatment group received intraperitoneal injections of GSPE powder dissolved in saline, whereas the control group received saline alone. Tail volume was measured periodically to monitor edema progression. Lymphatic and blood flow were assessed using near-infrared fluorescence indocyanine green lymphangiography (NIRF-ICGL) and laser Doppler flowmetry imaging (LDFI), respectively. Histological analysis was conducted using hematoxylin and eosin (H&E) staining.

**Results:**

The treatment group demonstrated a significant reduction of edema in the tail compared to the control group. NIRF-ICGL revealed improved lymphatic drainage, while LDFI analysis indicated enhanced blood perfusion in GSPE-treated animals. Histopathological examination showed reduced extracellular matrix deposition and fewer lymphatic abnormalities in the treatment group, suggesting mitigation of tissue fibrosis and lymphatic dysfunction.

**Discussion:**

These findings highlight the therapeutic potential of GSPE in treating secondary lymphedema. The observed improvements in lymphatic drainage, tissue perfusion, and histological features suggest that GSPE may exert beneficial effects beyond its established role in venous insufficiency. Considering the current lack of effective pharmacologic therapies for SLE, GSPE represents a promising candidate for future clinical applications. Further studies are warranted to validate its efficacy and safety in human subjects.

## Introduction

Cancer is not only challenging to treat, but the complications arising after the treatment process also significantly impact cancer survivors’ quality of life. With the global rise in cancer incidence and survival rates, diagnosing and managing complications arising from cancer treatment has become a critical focus in cancer care (1). Secondary lymphedema (SLE) is one of the most common post-treatment complications faced by many cancer survivors in particular. SLE refers to a chronic condition with localized interstitial fluid accumulation and subsequent complications caused by the physical disruption or dysfunction of lymphatic circulation. Acquisitive factors such as lymphatic injury, infection, or inflammation are the underlying causes of SLE, but surgical procedures (lymph node dissection) and radiation therapy associated with cancer treatment are the most significant causes. An estimated 140–250 million people are affected globally by lymphedema (LE), and SLE associated with cancer treatment accounts for the majority of these cases (2, 3). Although significant progress has been made in the prevention and treatment of breast cancer in recent years, its incidence and mortality rates remain unacceptably high (4, 5). Therefore, among patients with SLE, breast cancer-related LE accounts for the largest patient population worldwide and shows a strong clinical association.

Despite being the most critical condition to manage in post-cancer treatment care, SLE still lacks an established cure. Because of this, developing drugs for treating SLE is an emerging area of research. Several leading research groups are testing new substances for the development of medicines for SLE through preclinical and clinical studies (6–9), with grape seed proanthocyanidin extract (GSPE) emerging as one of the key candidates. Similar to sclareol, a potent anti-inflammatory derived from plants, the polyphenolic compounds abundant in GSPE are known to contribute to its antioxidant/anti-inflammatory activity and vascular-stabilizing effects (10, 11). In particular, proanthocyanidins in GSPE are known to stabilize capillary walls and reduce permeability, thereby preventing fluid leakage into tissues and improving the condition of the vascular wall extracellular matrix (12–16). These effects of GSPE may alleviate some of the pathological processes in SLE by mitigating oxidative stress and inflammatory responses (10, 17). However, the current literature lacks direct evidence evaluating the efficacy of GSPE specifically for SLE management. While its general health benefits are reported in the vascular system (16, 18), further research is necessary to establish its role and effectiveness in the lymphatic system as a treatment option for SLE. In this study, a tail LE animal model was used to evaluate the therapeutic effects of GSPE on SLE focusing on improving vascular and lymphatic circulation.

## Methods

### Study preparation and design

All procedures and experiments involving animals were reviewed and approved by the Institutional Animal Care and Use Committee (IACUC) of Asan Institute for Life Sciences, Asan Medical Center (approved <ns/>: 2023-30-142). The IACUC abides by the Institute of Laboratory Animal Resources (ILAR) and Animal Research: Reporting of *In Vivo* Experiments (ARRIVE) guidelines of The National Centre for the Replacement, Refinement and Reduction of Animals in Research (NC3Rs). The fifteen Sprague–Dawley rats of males weighing 250 – 300 g were used for this study. In the animal model, sexual differences were not considered because surgical methods induced the SLE condition. Before the surgery, the animals were allowed to approach water and ad libitum freely under stable humidity and temperature conditions. Twelve animals underwent the surgical procedure, while the remaining three animals were observed in their normal state without the surgery.

After the surgery, the animals underwent a one-week recovery period during which the formation of LE was monitored. The LE animals received daily medication for two weeks starting from the first week. In the experimental group (medication group), GSPE compound powder (HL189) provided by Hanlim Pharmaceutical company in the Republic of Korea was mixed in saline and administered intraperitoneally at a dose of 100 mg/kg, adjusted to each animal’s weight, with a total volume of 1 ml per injection. The control group received the same volume of saline. The GSPE dosing was determined based on previous studies (19). Swelling in the tail was followed weekly, and the lymphatic and blood flow in each group’s tail were evaluated at week 8. After the evaluation, the animals were sacrificed and tail tissues were harvested for histological examination (**Supplementary Figure 1**).

### Production of tail LE animal models

The animals were limited to fed for 1 week preoperatively, and the same investigator performed the procedures related to animal experiments. They were anesthetized with isoflurane gas in a concentration of 4% and Tiletamine/Zolazepam (Zoletil, Virbac, France) mixed with Xylazine (Rumpun, Bayer Korea, Republic of Korea) by volume ratio 5:1. After anesthetization, 0.05-mL Evans blue (Sigma-Aldrich, St. Louis, MO) solution (blue dye; 30 mg/mL solution in 0.9% saline) was subcutaneously injected into the end of tail to identify lymphatic vessels (LVs). After disinfecting the surgical area with 75% ethyl alcohol, the skin along the circumference of the dermis layer is removed by approximately 2 mm in width at a point 20 mm distal from the base of the tail. Within the excised skin gap, two collecting LVs were located on both sides of veins in the lateral directions. These vessels were identified using blue dye, and also using near-infrared indocyanine green lymphangiography (NIRF-ICGL) with indocyanine green (ICG) as the contrast agent (**Supplementary Figure 2A**). An electrocautery device (Bovie^®^; Symmetry Surgical Inc., Antioch, TN) was used to carefully cauterize the LVs and skin incision edges circumferentially without injuring the vein or artery (If vascular damage occurs, the tail may necrotize) (**Supplementary Figure 2B**). After the procedure, Ketoprofen (1 mg/kg; SCD Ketoprfofen Inj., SamChunDang Pharm, Republic of Korea) was injected intramuscularly immediately. One week post-surgery, scarring forms around the circumference of the tail following the skin incision line accompanied by observable swelling. During this period, necrosis and infection of the wound were monitored closely to assess swelling formation. Animals that failed to develop LE form were excluded from the study and sacrificed.

### Evaluation of edema volume in the animal model

The edema (swelling) formation was evaluated by analyzing the diameter ratio between the 5 mm proximal region (normal part) and the 5 mm distal region (LE part) relative to the incision line (**Supplementary Figure 3**). Although the tail of the SD rat is conical in shape and tapers distally, the tail is generally very long (often exceeding 200 mm) compared to the distance between measured points of each part (10 mm apart). Because of this, the tail diameter ratio in the normal state was nearly 1. Furthermore, comparing the normal part with the LE part induced by surgery could minimize errors arising from inter-individual variability. This analysis was conducted using photographic analysis software, ImageJ software (ImageJ 1.48 v, http://rsbweb.nih.gov/ij/; NIH, Bethesda, MD, USA).

### Evaluation of lymphatic flow using NIRF-ICGL

A representative modality for non-invasive lymphatic imaging is NIRF-ICGL. This technique involves injecting an indocyanine green (ICG) contrast agent into the lymphatic system and detecting the fluorescence emitted by the agent. Unlike blood, which is colored due to red blood cells, the lymphatic system is colorless and transparent, therefore, this technique is highly effective for visualizing lymphatics. We used a customized imaging system with 4.2-watt high-powered LEDs (730 nm peak, LST1-01G01-FRD1-00; Opulent Americas, Raleigh, NC) and a 2-inch bandpass filter (FF01-832/27-50-D; Semrock, West Henrietta, NY) to visualize lymphatic vessels in the tail. To visualize LVs in the tail, 2 μL of ICG solution (Diagno Green inj.; Daiichi Sankyo, Tokyo, Japan) mixed with 2.5 mg/ml of bovine serum albumin (Sigma-Aldrich, Saint Louis, MS) was injected into the tip of the tail. These conditions were established as optimal for obtaining high-quality lymphatic imaging in previous studies (20, 21). The injection site was gently massaged to absorb the ICG dye into the LVs. Images were acquired 30 minutes after the ICG injection.

### Evaluation of blood flow using laser Doppler imager

A laser Doppler flowmetry imager (LDFI), moorLDI2-IR (Moor Instruments Ltd, Axminster, United Kingdom), was employed to evaluate the changes in blood flow associated with the onset of LE in the tails of animal models and the effects of GSPE administration. This system with a spatial resolution of 100 microns used raster scanning with 785 nm wavelength light to visualize relative flux in blood flow under the skin by utilizing red blood cells as reflectors. The extent of blood flow is defined through ‘flux’, which is a quantity proportional to the product of the average velocity of the blood cells and their number concentration. We performed scans over an area of 2.5 cm × 2.5 cm at a resolution of 512 × 512 pixels with the imaging system positioned at a height of 20 cm above the surface. LDFI and NIRF-ICGL imaging were performed on animals in the same fixed position with only the equipment changed. It allowed lymphatic flow and blood flow could be compared at identical locations of the same animals. We composited these images for comparative analysis.

The blood flow flux was represented as relative values and visualized using a rainbow color map (red: higher flow, blue: lower flux). Additionally, a fixed laser beam in the LDFI scan allowed real-time acquisition of blood flow graphs (single point measurement). These measurements were conducted for the control and medication groups enabling a direct comparison of blood flow dynamics.

### Histological analysis

Animals from the control and medication groups were sacrificed after the whole follow-up period, and the tails were harvested with 10 mm taken proximally and 10 mm distally from the incision line as the central reference point (total of 20 mm). The harvested tissues were fixed with 4% formaldehyde solution and rinsed with tap water to remove the fixative for 2 hours. To examine the cross-sectional tissue of the tail, the tissue including the bone was decalcified using EDTA (ethylenediaminetetraacetic acid; Sigma-Aldrich, Saint Louis, MS) for 7 – 8 days. The tissues were cleared in xylene using a tissue processor (Excelsior ES, Thermo Fisher Scientific, Waltham, MA) and embedded into paraffin blocks sectionally using an embedding station system (EG1150H, Leica, Wetzlar, Germany). The paraffin blocks were cut into 5-µm-thick sections on a rotary microtome (RM2255, Leica, Wetzlar, Germany) at positions 5 mm proximal (normal part) and 5 mm distal (LE part) from the incision line. The blocks were stained with hematoxylin and eosin (H&E). Images were obtained from the magnified 10× images using microscopy (BX40 type; Olympus, Tokyo, Japan). The dermal thickness ratio (normal part/LE part) was averaged at six randomly selected regions. The thickness of blood vessels and the size of lymphatic vessels were evaluated at anatomically identified locations based on the bone as a measurement reference point in **Supplementary Figure 3** using ImageJ software.

### Statistical analysis

The data errors are presented as the mean and standard deviation of the mean. The statistical analyses were performed using GraphPad Prism 10 (GraphPad Software Inc., CA, USA) and Microsoft Excel 2019 (version 2111, Microsoft Corporation, CA, USA). The t-test assuming equal variances and one-way ANOVA were used and * p-value < 0.05 was considered to indicate a statistical significance.

## Results

### Difference of edema volume change in both group

**Figure 1:**
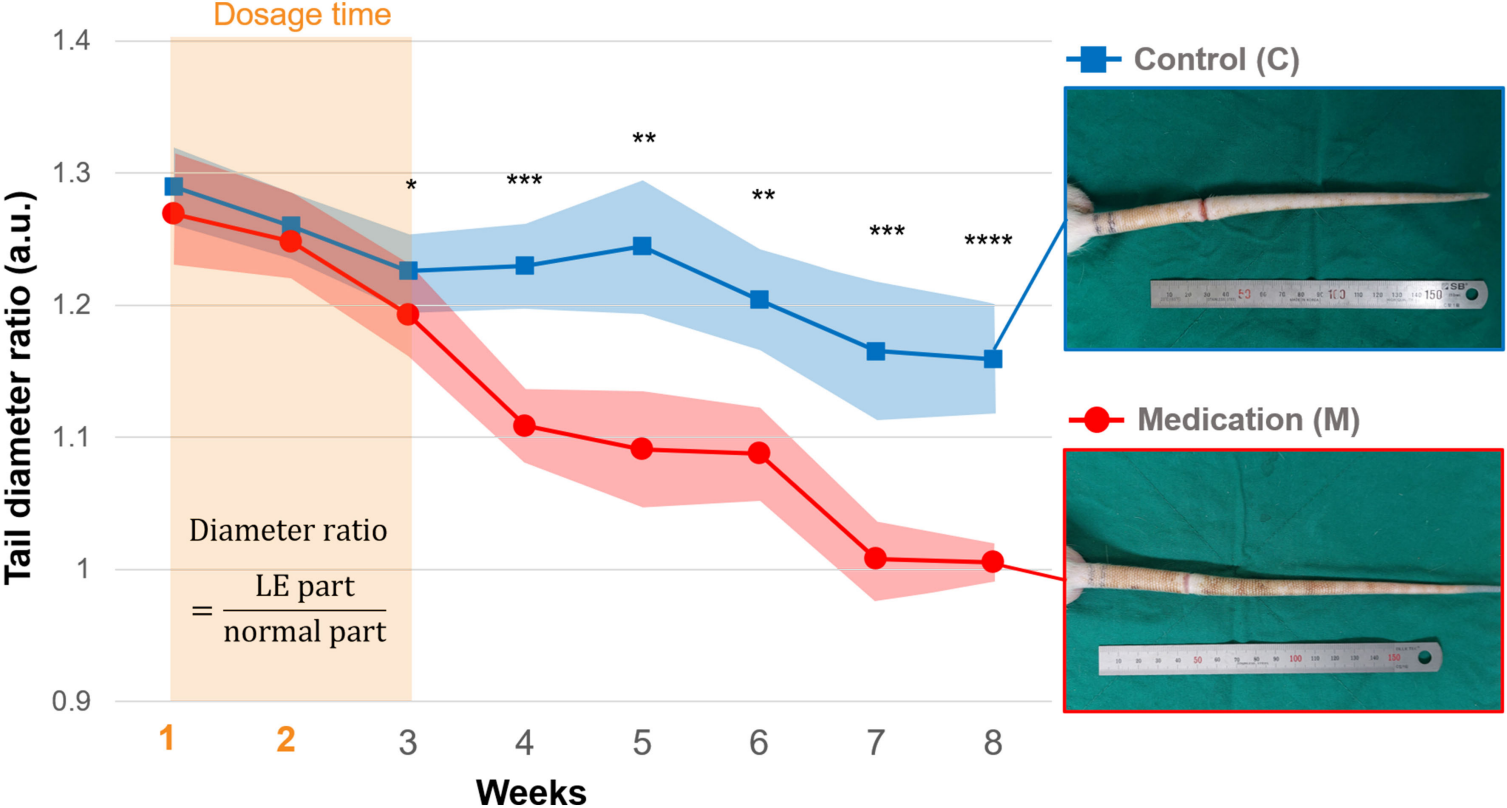
The tail diameter ratio of the control and medication group during the follow-up period, and photos of tails in the 8th week. From the 1st week, daily injections of saline (control group) and GSPE solution (medication group) were administered for 2 weeks. Significance was assessed by using Student’s t-test with p-value. Adjusted significance levels are denoted as p < 0.05 (*), p < 0.01 (**), p < 0.005 (***), and p < 0.001 (****).

The tail diameter ratio increased to approximately 1.3 in both the control and medication groups following the formation of the LE condition. There were no notable differences between the two groups during the two-week GSPE administration period. However, the difference between the two groups began to appear in 3rd week following the finish of the drug administration period. From the 4th week, a significant decrease in the diameter ratio was observed in the medication group (**Figure 1**). The significant p-values were 0.028 (*), 0.004 (***), 0.010 (**), 0.019 (**), 0.004 (***), and 0.0002 (****), respectively.

### Lymphangiogenesis and change of lymphatic drainage by GSPE administration

**Figure 2:**
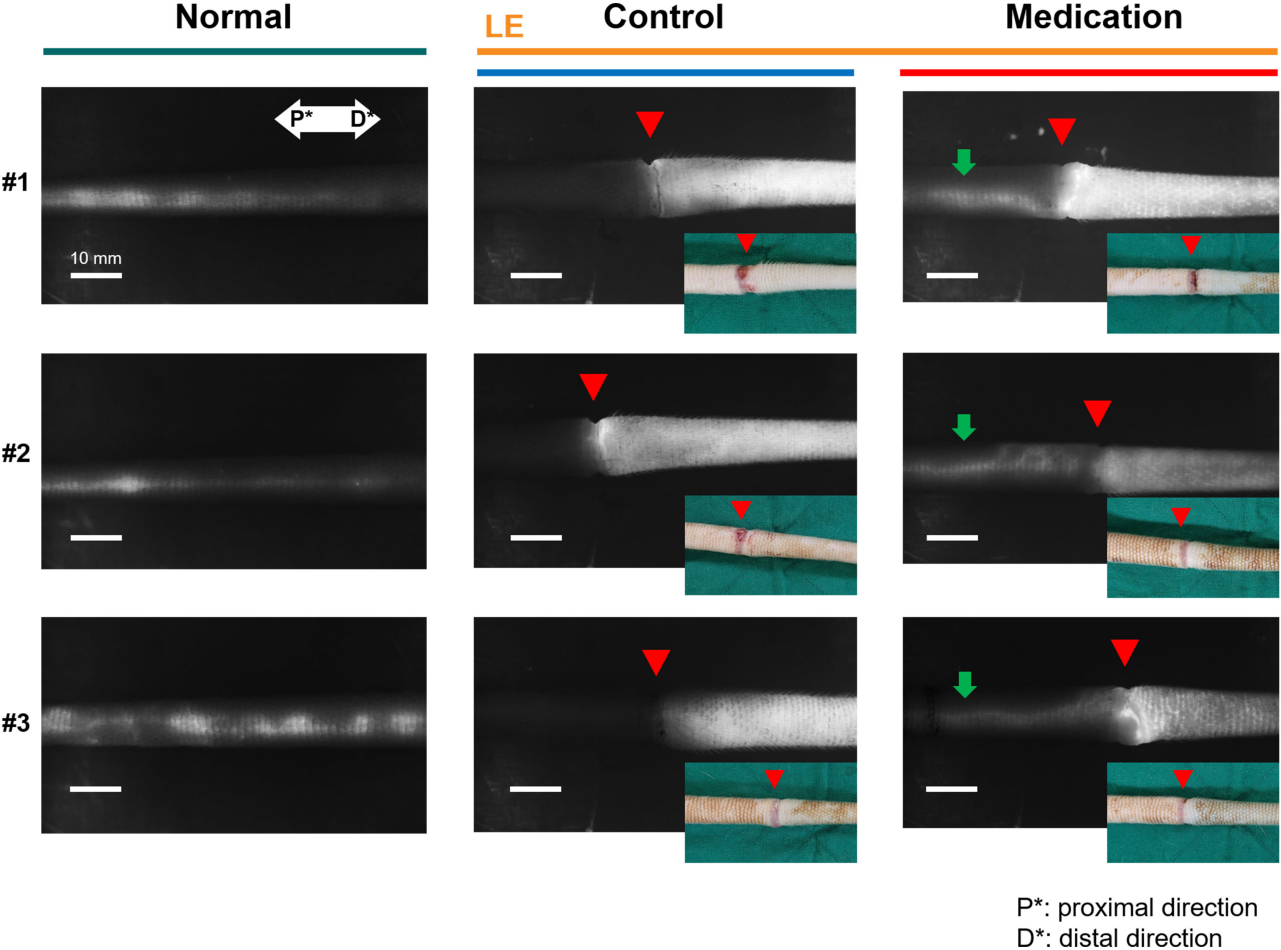
Representative NIRF-ICGL images of the normal group, (LE) control group, and (LE) medication group. The red triangle indicates the incision line, corresponding to the location marked by the red triangle in the inset tail photo. The green arrow in the medication group image highlights lymphatic flow extending proximally beyond the incision line. P* and D* represent the proximal and distal parts of the tail, respectively.

**Figure 2** presents representative NIRF-ICGL images highlighting the differences in lymphatic drainage among normal conditions, and control and medication groups under SLE conditions. In NIRF-ICGL, the lymphatic drainage in the normal condition was observed in a linear pattern along the LVs located near the veins. In the control group, lymphatic drainage was restricted at the incision line (red triangle) resulting in lymphatic fluid spreading diffusely across the entire distal tail. This demonstrates that lymphatic drainage in the distal region of the incision line failed to move proximally and lymphatic fluid was accumulated in the interstitial tissues. Conversely, although a diffuse pattern was still observed in the distal tail, lymphangiogenesis, regeneration of lymphatic vascular endothelial cells (22, 23), was evident and the new LVs allowed lymphatic drainage to bypass the incision line and extend proximally as indicated by green arrows in the medication group.

**Figure 3:**
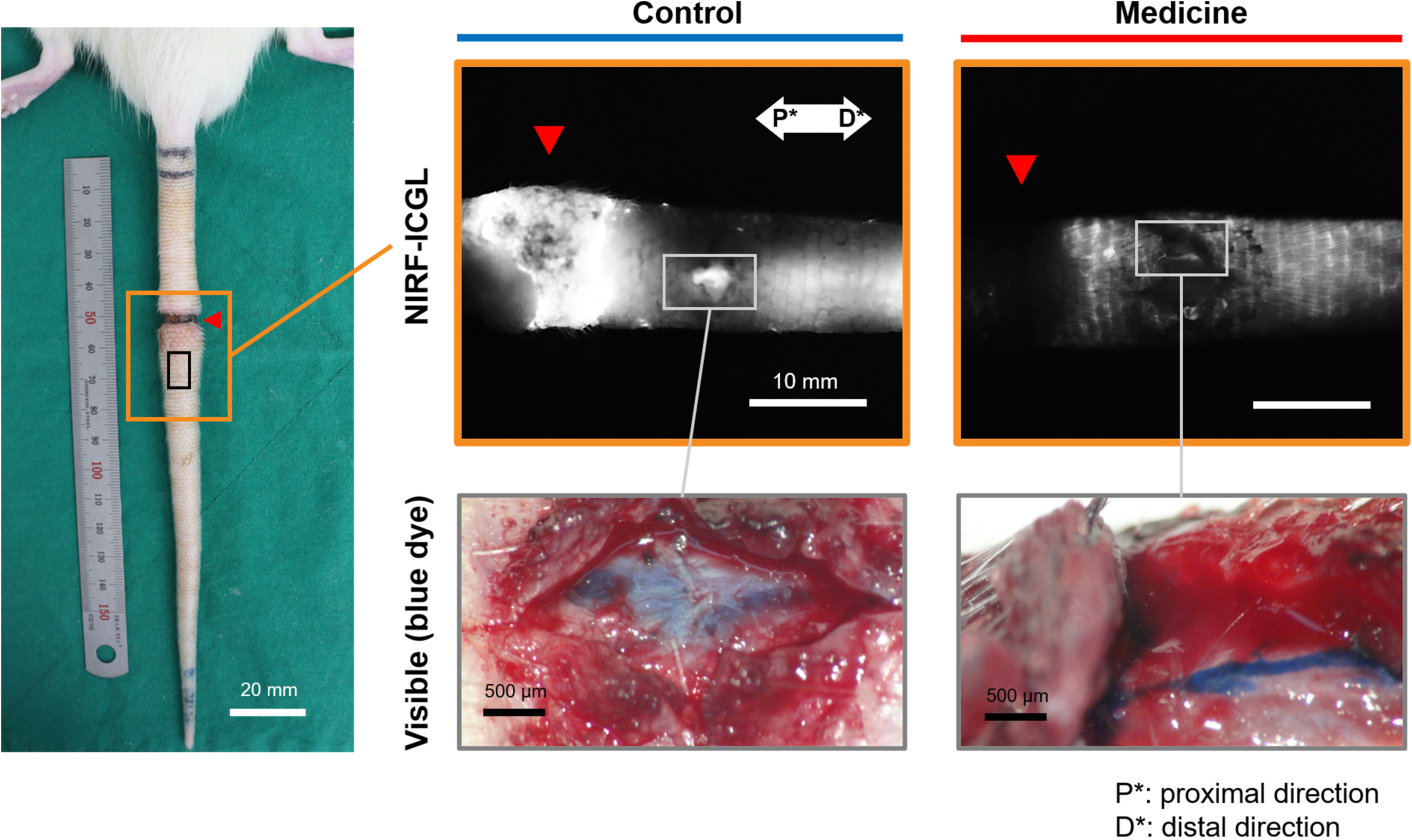
NIRF-ICGL and visible images (with blue dye) of exposed the LVs obtained after removing the skin of the LE part, where ICG had diffused in the control and medication groups. The red triangle indicates the location of the original incision line. P* and D* represent the proximal and distal parts of the tail, respectively.

The condition of LVs in the LE part was challenging to observe non-invasively in both the control and medication groups due to the dispersion of ICG contrast agents across the skin. To address this, blue dye was re-administered using the same method employed during model production, and a small skin incision was made 5 mm distal to the incision line (red triangle in **Figure 3**) to directly observe the LVs. **Figure 3** presents LVs observed under the skin in the LE parts of both groups using visible imaging with (Evans) blue dye and NIRF-ICGL imaging with ICG dye. The structure of LVs in the control group was difficult to distinguish due to fibrotic deposits encasing the LVs. In contrast, the medication group exhibited clear surrounding tissue and distinctly visible LVs structure.

### Change of blood flow flux by GSPE administration

**Figure 4:**
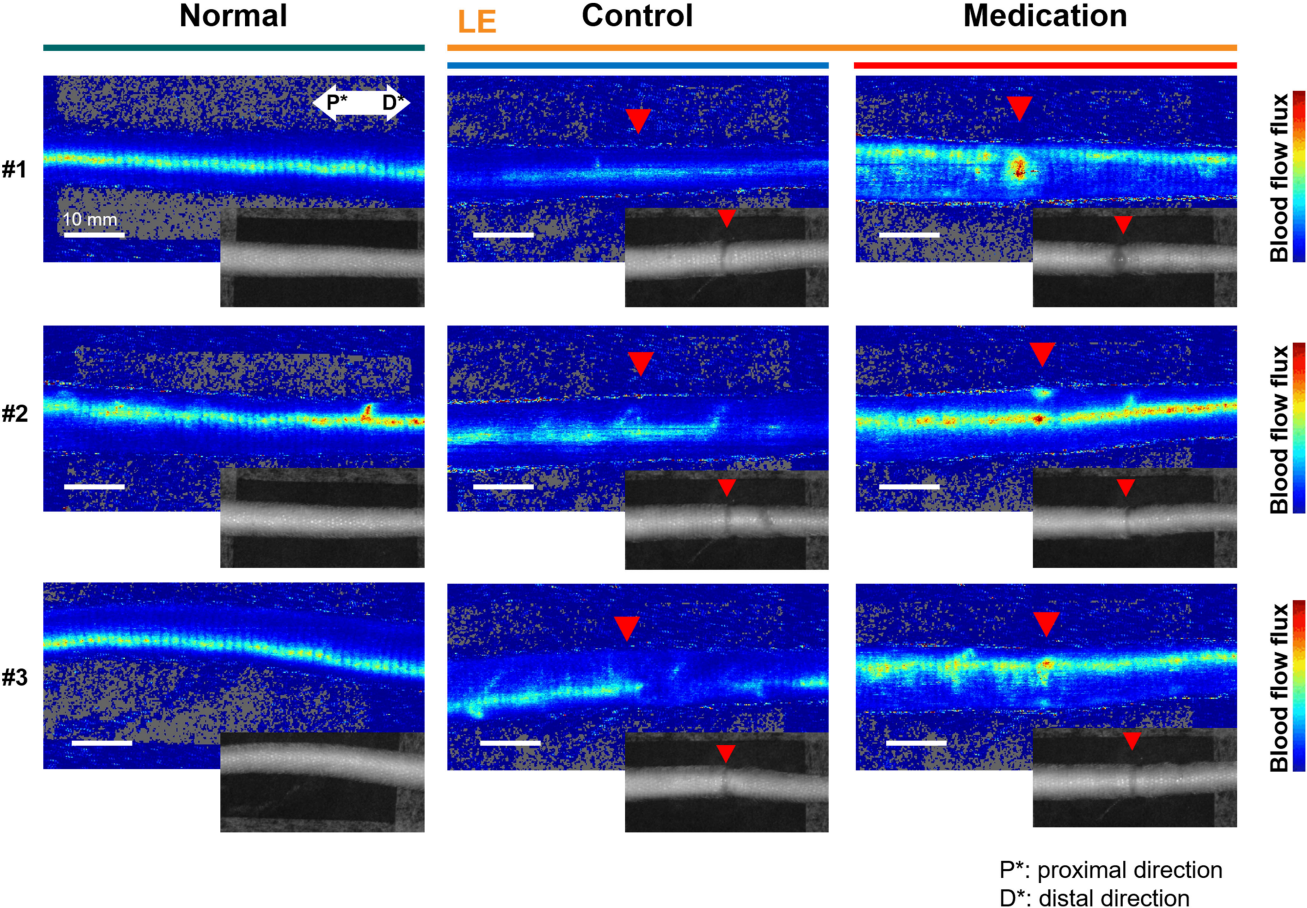
Representative LDFI images of the normal, (LE) control, and (LE) medication groups. The red triangle indicates the incision line, corresponding to the location marked by the red arrow in the inset tail photos. The blue color represents weak flux transitioning to red for stronger flux. P* and D* represent the proximal and distal parts of the tail, respectively.

In the surgical procedure to induce SLE condition, damage to the artery or vein can lead to tissue necrosis, and it was prevented in the formation of a proper model. Thus, venous blood flow remains present in both normal and SLE conditions. However, the SLE condition caused notable changes in blood flow flux. **Figure 4** presents representative blood flow flux images obtained through LDFI with the inset showing the corresponding photo images. As shown in **Figure 4**, the SLE condition in the control group reduced overall blood flow flux beyond the incision line (red triangle). In contrast, the medication group treated with GSPE exhibited an approximately twofold increase in overall blood flow flux compared to the control group. The relative blood flow flux intensity was an average of 327.1 in the medication group versus 156.2 in the control group.

**Figure 5:**
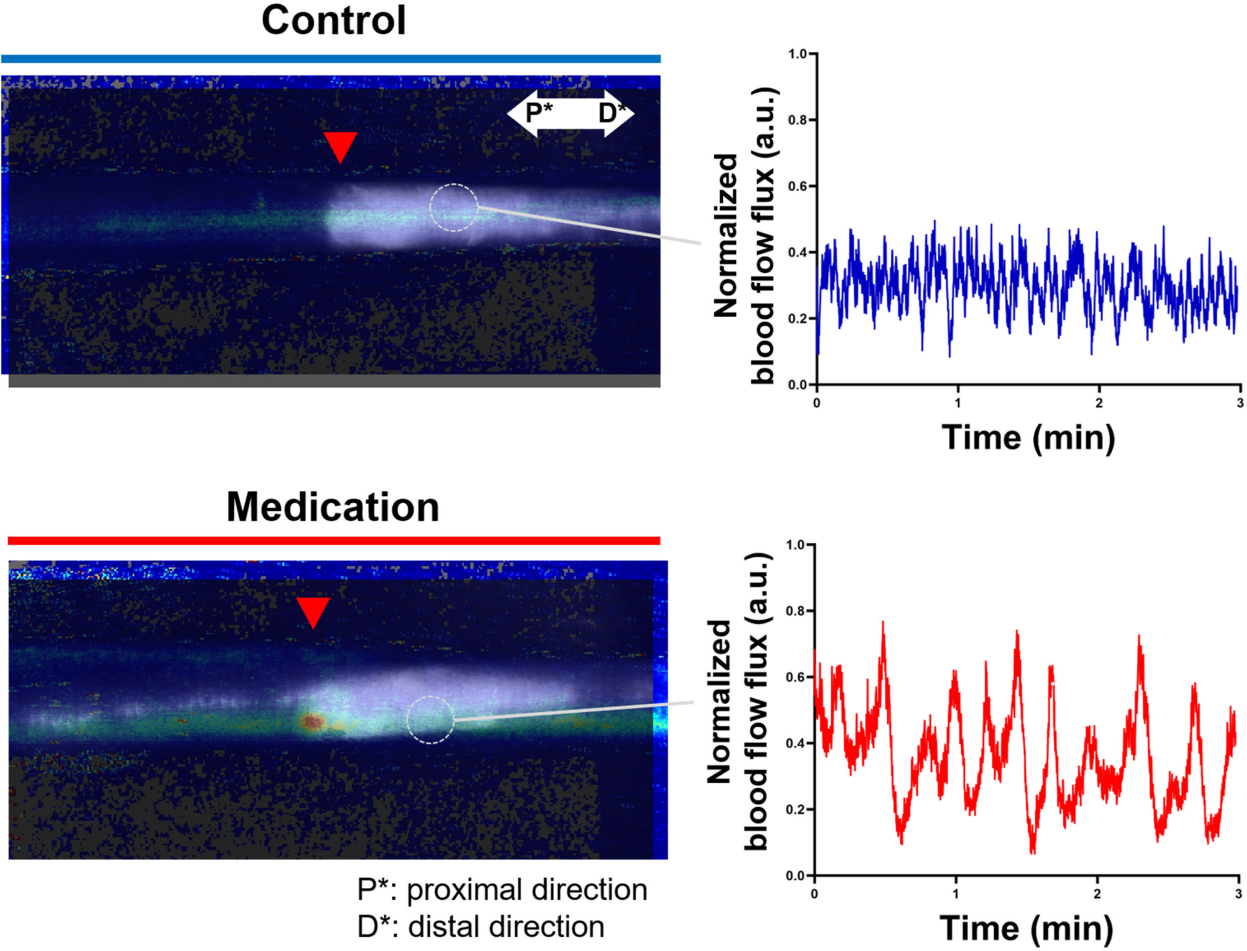
Composite images of LDFI and NIRF-ICGL for the control and medication groups along with a dynamic blood flow flux measured during 3 minutes at a single point on the vein in the LE part. The time-dependent graph illustrates blood flow dynamics. The flux values were normalized based on the minimum and maximum flux values in the measurement. P* and D* represent the proximal and distal parts of the tail, respectively.

We dynamically analyzed the differences in blood flow changes between the control and medication groups by observing flux pulse variations over time at a single point on the vein in the LE part. **Figure 5** presents the composite image of LDFI and NIRF-ICGL for tails in the control and medication groups. Measurements were taken at the vein located 5 mm distal to the incision line (red triangle) within the LE part for 3 minutes, and the normalized intensity values of dynamic blood flow flux were plotted. As shown in the graph of **Figure 5**, the medication group exhibited a significantly bigger and cleaner waveform with approximately twice the peak-to-peak pulse intensity compared to the control group (control: ~0.4, medication: ~0.75).

### Dermis thickness, blood vessel wall thickness, and area of LVs

**Figure 6:**
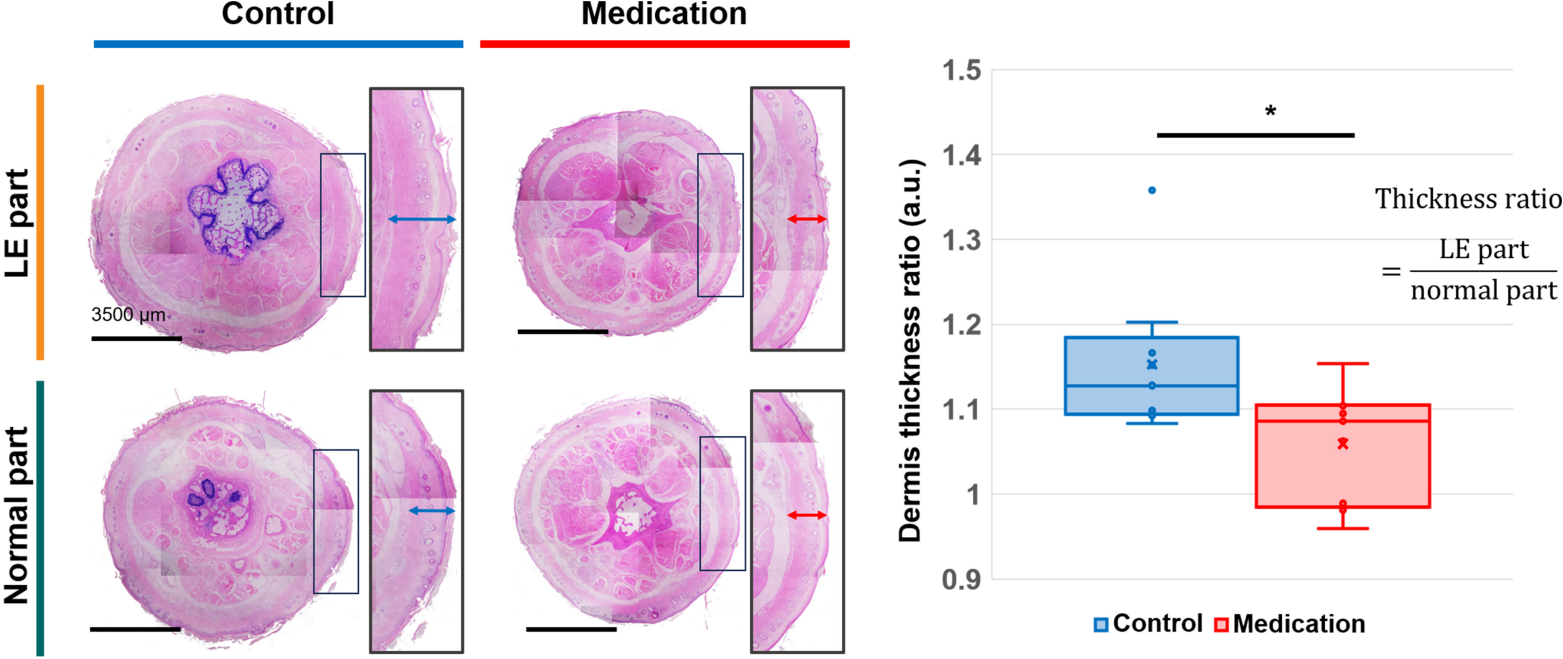
H&E-stained cross-sectional images of normal part and LE part tail tissues from the control and medication groups, and a comparison of the averaged dermis thickness ratio between the two groups. Adjusted significance is denoted as p < 0.05 (*) using Student’s t-test.

To directly evaluate tissue changes induced by GSPE administration in SLE conditions, we measured the dermis thickness in both the control and medication groups. **Figure 6** presents the cross-sectional images of the normal part and LE part in the tails, as well as the ratios of dermis thickness between these parts for each group. In the control group, SLE resulted in increased dermis thickness in the LE part. However, the medication group exhibited a significantly reduced dermis thickness compared to the control group. The p-value was 0.021 (*). There was no significant difference among the normal parts of the control group and medication group, and the LE part of the control group (p-value = 0.125), but the LE part of the medication group had a significant difference with the normal parts of these groups (p-value < 0.0001).

**Figure 7:**
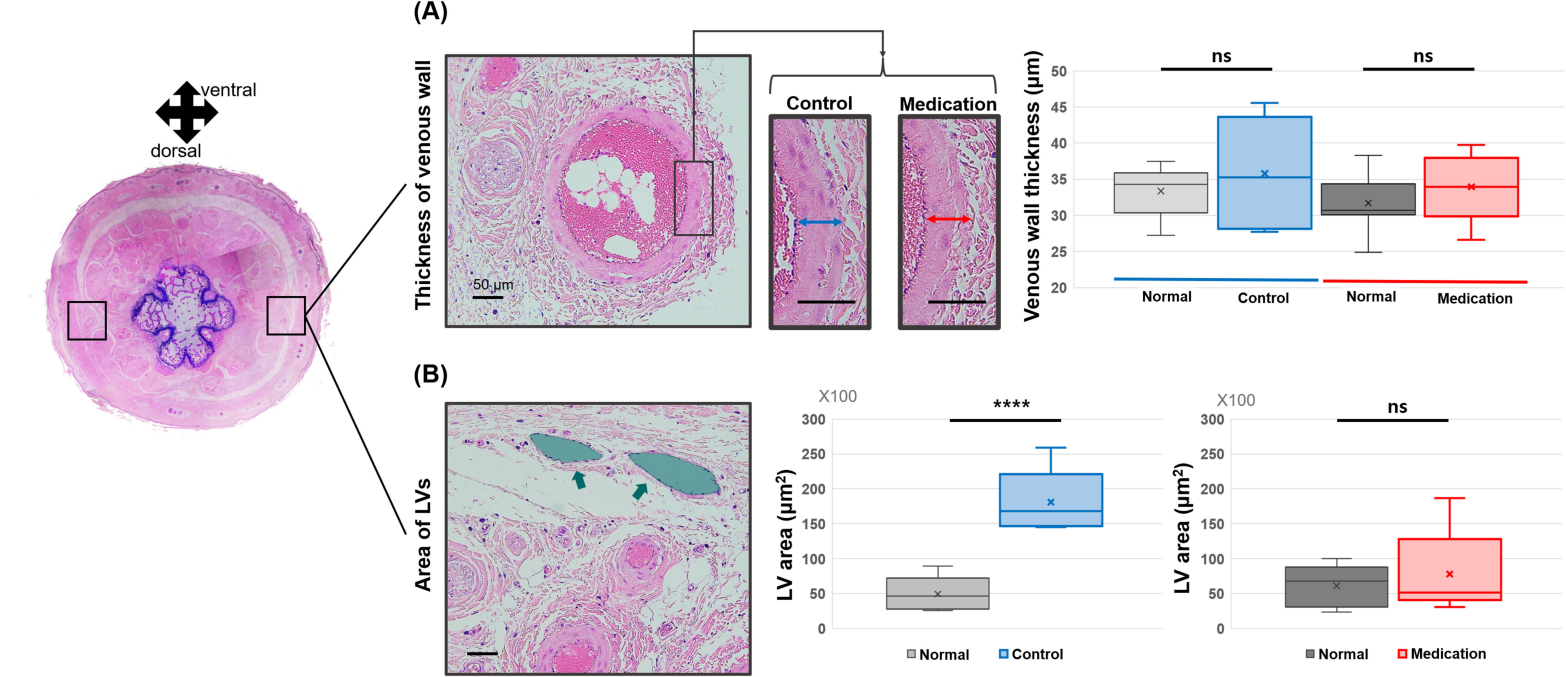
Comparison of **(A)** vessel wall thickness in lateral veins and **(B)** LV (green arrow and area) area between the control group and the medication group. Significance was assessed by using Student’s t-test with p-value. Adjusted significances are denoted as p > 0.05 (ns) and p < 0.001 (****) using Student’s t-test.

Next, we examined the histological changes in veins and LVs. Measurements of the venous wall thickness near LVs revealed no significant differences between the control and medication groups compared to the normal condition (**Figure 7A**). No significant difference was found among LE parts of the control group and medication group, and normal parts of these groups (p-value = 0.430). In contrast, notable differences were observed in the LVs. The control group exhibited a significant increase in the average cross-sectional area of LVs, indicative of dilation of LVs. However, in the medication group, the LV morphology and size were comparable to the normal condition (**Figure 7B**). The p-values were 0.0005 (****) and 0.606 (ns), respectively. A one-way ANOVA revealed a significant difference in the area of LVs among normal parts of the two groups and the control group (p-value = 0.0001), and no significant among normal parts of the two groups and the medication group (p-value = 0.585), respectively.

## Discussion

GSPE which is rich in proanthocyanidins demonstrates significant potential in improving the conditions of venous and lymphatic function. These improvements may be derived from its antioxidant, anti-inflammatory, and endothelial-enhancing properties, which directly influence the structural and functional integrity of these circulatory systems. The proanthocyanidins mitigate oxidative stress by neutralizing reactive oxygen species, and this leads to improved nitric oxide production to optimize blood flow (24). Furthermore, the anti-inflammatory properties of GSPE reduce the expression of cytokines like TNF-α and IL-6, and mitigate chronic inflammation. In previous research, GSPE was anticipated to decrease edema formation (25), oxidative stress markers, such as malondialdehyde (26–28), and inflammatory cytokine levels (29–32). In particular, the molecular and biological mechanisms of GSPE have been continuously elucidated through various cellular and animal studies. Chao, CL et al. demonstrated in a cell-based study that GSPE improves TNF-α-induced inflammatory status (29), and Vaid, M et al. showed in a UV-induced inflammatory mouse model that GSPE suppresses UV-induced immunosuppression by stimulating CD8(+) effector T cells and reducing regulatory CD4(+) T cells (30). Dimitriu, T et al. demonstrated that oral administration of GSPE in a periodontitis rat model led to a reduction in oxidative stress, inflammation, and atherosclerosis (27). Similarly, Belviranli, M et al. showed that GSPE attenuated exercise-induced oxidative stress in a rat model, suggesting that GSPE’s effects extend beyond specific cells to systemic physiological processes (26). These cell-based and animal studies across diverse diseases and target organs collectively suggest that the anti-inflammatory and antioxidant effects of GSPE are not confined to a single pathology or site, but rather exert broad-spectrum therapeutic potential. In addition, Entelon^®^ (Hanlim Pharm Co. Ltd, Seoul, Republic of Korea) is an available medicine for the improvement of symptoms related to intravenous lymphatic dysfunction as GSPE in South Korea. In summary, based on existing findings, GSPE will reduce swelling, enhance the functionality of blood vessels and LVs, and diminish inflammation under SLE conditions.

In this study, we demonstrated through preclinical research that GSPE may effectively alleviate SLE and its therapeutic effect occurred by these anticipated effects using the SLE tail animal model. The reduction in swelling in the medication group began to show following the GSPE administration, and by the 8th week, the medication group approached near normal conditions (close to 1). This observation aligns with the histological results for the dermis thickness ratio in the 8th week. The observed reduction in swelling appeared to result from improved lymphatic drainage. In the NIRF-ICGL imaging, the medication group exhibited lymphangiogenesis extending beyond the incision line. This might have occurred due to enhanced endothelial integrity, reduced permeability, and minimized fluid leakage according to previous studies (33, 34). However, based on our findings about the condition of the tissue surrounding the LVs under the skin in the LE part as shown in **Figure 3**, it is evident that the anti-fibrotic/anti-inflammation effects of GSPE played a significant role. Although the dermal backflow was not completely resolved, the medication group demonstrated well-preserved functionality and structural integrity of the collecting LVs under the skin, which was absent in the control group.

Another notable result was the improvement in venous flow and function induced by GSPE administration. We observed a decrease in blood flow flux in the tail veins under the SLE condition (control group) compared to the normal condition. The reduced blood flow flux with impaired lymphatic drainage in SLE conditions can be explained by several mechanisms. First, increased interstitial pressure in the LE-affected tissue might compress the surrounding venous structures, thereby reducing blood flow flux. Second, poor lymphatic drainage is associated with venous congestion (35, 36), which may also contribute to insufficient blood flow flux and result in overall impairment of hemodynamics. However, the administration of GSPE was shown to increase blood flow flux by using LDFI in the medication group. Previous studies have demonstrated that low-intensity pulsed ultrasound stimulated blood flow flux to relieve SLE condition (37). The suppression of fibrotic deposition around the veins and LVs in the medication group may have reduced mechanical resistance and restored vascular integrity leading to enhanced hemodynamic performance.

Despite hemodynamic improvement in the LDFI results, histological analysis revealed no structural changes in veins in the control and medication groups. It is possible that the 8-week follow-up period was insufficient to induce significant structural changes in the veins even if the blood flow flux was changed. However, the LV dilation differed significantly between the control and medication groups. In the control group, LV dilation was maintained even at the 8-week, whereas it was nearly resolved in the medication group. Prolonged LV dilation can lead to chronic lymphatic insufficiency exacerbating LE progression (38–40). The histological findings support the notion that GSPE administration may mitigate this vicious cycle and contribute to improved lymphatic function.

This study has several limitations. First, it did not present direct functional enhancements in LVs such as lymphatic contraction patterns. Measuring *in vivo* lymphatic contraction signals in the rodent tail presents inherent limitations, while valuable for observing lymphatic structure and macroscopic fluid transport. The tail model is anatomically restricted due to the superficial positioning of LVs and the scales covering the surface of the tail skin, impeding precise quantification of lymphatic contractile activity. Furthermore, the tail lymphatics exhibit low amplitude of spontaneous contractions, which makes the detection and differentiation of true lymphatic contractile signals from background noise particularly challenging (41). Second, the exact mechanisms behind the increase in blood flow flux following GSPE administration were not elucidated. Although previous studies have reported the effects on blood flow improvement induced by GSPE administration (16, 28), additional molecular and biochemical investigations are required to provide detailed evidence. Our findings indicate promising therapeutic effects of GSPE in the SLE model, but we acknowledge that these results are derived solely from animal experiments. The extensive translational studies will be necessary to assess safety and efficacy in humans. Lastly, the potential differences in outcomes between intraperitoneal and oral administration of GSPE were not explored.

## Conclusion

In this study, we demonstrated the therapeutic potential of GSPE administration through preclinical research focusing on its role in improving venous flow and lymphatic circulation, which could contribute to alleviating SLE. This study demonstrates *in vivo* that GSPE administration in an SLE animal model leads to reduced edema, enhanced lymphatic drainage, and improved hemodynamics. Although further research is needed to clarify the underlying mechanisms, determine optimal dosing, and assess safety, these preclinical results suggest the potential of GSPE to alleviate conditions in clinical SLE patients and provide a foundation for the development of GSPE-based therapeutic agents for SLE.

## Data Availability

The original contributions presented in the study are included in the article/**Supplementary Material**. Further inquiries can be directed to the corresponding author.
