# Supplementary material for: Grape seed proanthocyanidins improve lymphatic drainage and blood perfusion in secondary lymphedema models
Source: Front Oncol. 2025 Jun 6;15:1553090. doi: 10.3389/fonc.2025.1553090 (PMC12179177; doi:10.3389/fonc.2025.1553090)
Supplement: Supplementary file 1 [file DataSheet1.pdf]

Supplementary Materials

Grape Seed Proanthocyanidins Improve Lymphatic Drainage and Blood Perfusion in Secondary Lymphedema Models

Hwayeong Cheon, Bumchul Kim, and Jae Yong Jeon

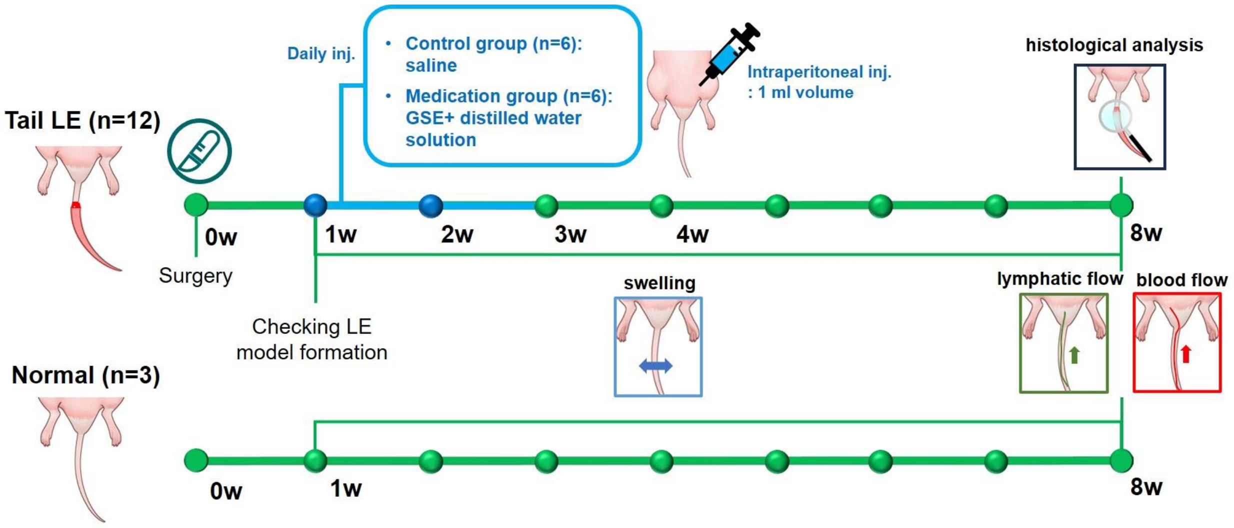

Suppl. Fig. 1. Research schedule of this study.

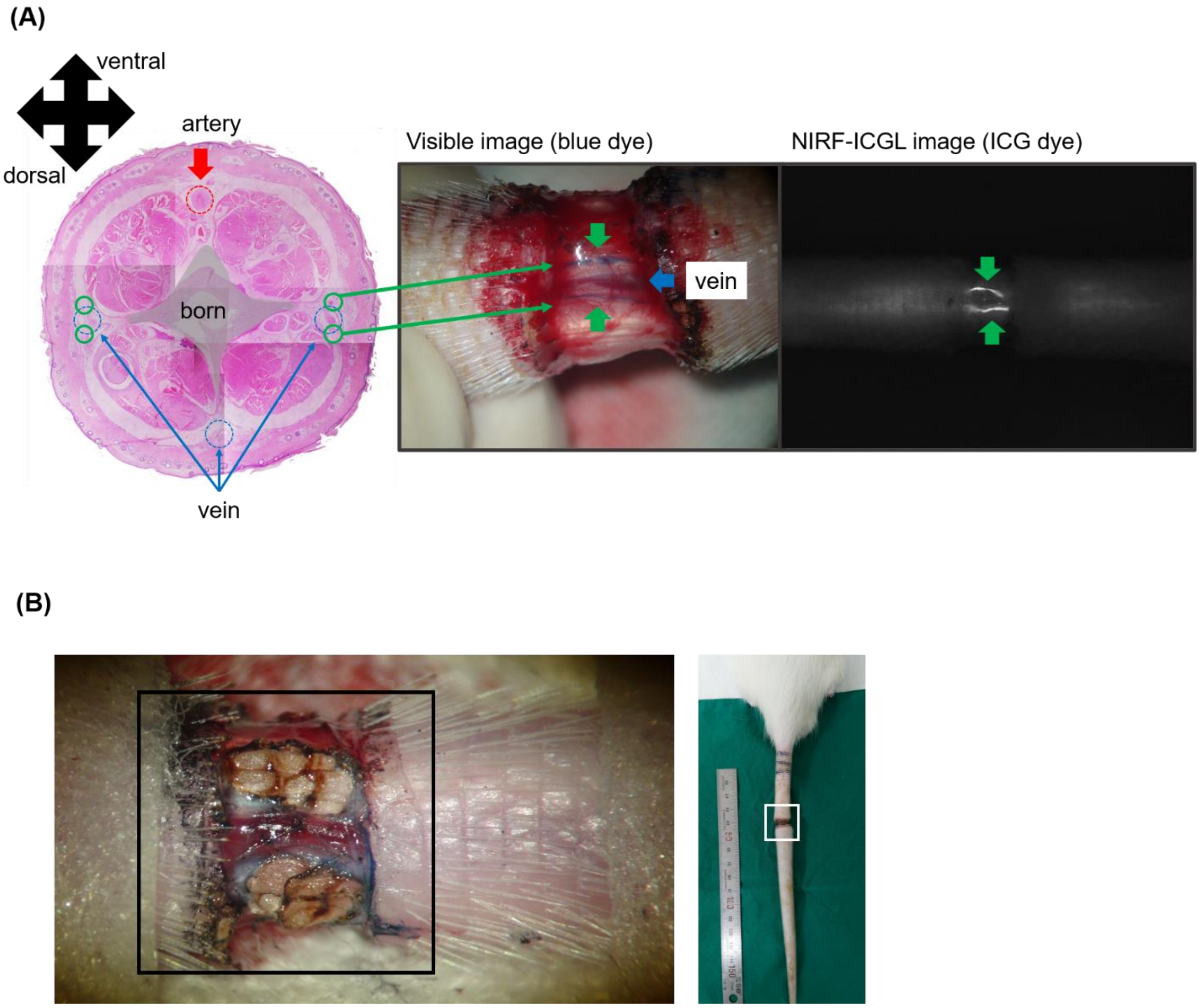

Suppl. Fig. 2. (A) Cross-sectional image of tail tissue using H&E staining, visible imaging with Evans blue dye, and NIRF-ICGL with ICG dye. LVs were identified near the vein on both lateral sides of the central bone. The LVs were positioned above and below the vein. **(B)** Image showing selective cauterization of collecting LVs near the vein without damaging the vein itself, and the appearance of a tail LE model post-surgery.

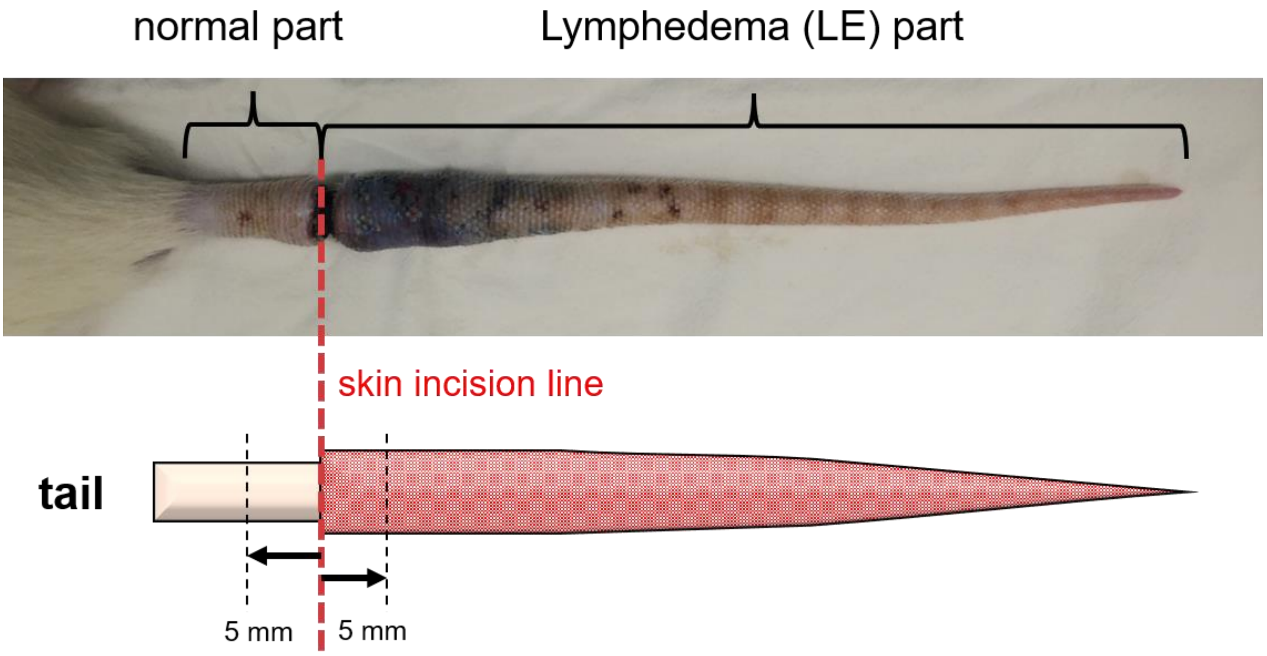

Suppl. Fig. 3. The normal part and LE part defined relative to the incision line, and measurement reference points (5 mm distances from the incision line in both the proximal, normal part, and distal, LE part, directions).
